# Using heart rate profiles during sleep as a biomarker of depression

**DOI:** 10.1186/s12888-019-2152-1

**Published:** 2019-06-07

**Authors:** Mysa Saad, Laura B. Ray, Brad Bujaki, Amir Parvaresh, Iryna Palamarchuk, Joseph De Koninck, Alan Douglass, Elliott K. Lee, Louis J. Soucy, Stuart Fogel, Charles M. Morin, Célyne Bastien, Zul Merali, Rébecca Robillard

**Affiliations:** 1 Sleep Research Unit, The Royal’s Institute of Mental Health Research, 1145 Carling Avenue, Ottawa, ON K1Z 7K4 Canada; 2 Department of Cellular and Molecular Medicine, University of Ottawa, 451 Smyth Road, Ottawa, ON K1H 8M5 Canada; 3 Sleep Clinic, Royal Ottawa Mental Health Centre, 1145 Carling Avenue, Ottawa, ON K1Z 7K4 Canada; 4 School of Psychology, University of Ottawa, 136 Jean-Jacques Lussier, Ottawa, ON K1N 6N5 Canada; 5 Department of Psychiatry, University of Ottawa, 1145 Carling Avenue, Ottawa, ON K1Z 7K4 Canada; 6 Centre d’étude des troubles du sommeil, Centre de recherche de l’institut universitaire en santé mentale de Québec, 2525 boulevard de la Canardière, Québec, QC G1J 2G3 Canada; 7 School of Psychology, Université Laval, 2325 rue des Bibliothèques, Québec, QC G1V 0A6 Canada; 8 CERVO Brain Research Centre, 2601 Chemin de la Canardière, Québec, QC G1J 2G3 Canada

**Keywords:** Heart rate variability, Autonomic nervous system, Major depressive disorder

## Abstract

**Background:**

Abnormalities in heart rate during sleep linked to impaired neuro-cardiac modulation may provide new information about physiological sleep signatures of depression. This study assessed the validity of an algorithm using patterns of heart rate changes during sleep to discriminate between individuals with depression and healthy controls.

**Methods:**

A heart rate profiling algorithm was modeled using machine-learning based on 1203 polysomnograms from individuals with depression referred to a sleep clinic for the assessment of sleep abnormalities, including insomnia, excessive daytime fatigue, and sleep-related breathing disturbances (*n* = 664) and mentally healthy controls (*n* = 529). The final algorithm was tested on a distinct sample (*n* = 174) to categorize each individual as depressed or not depressed. The resulting categorizations were compared to medical record diagnoses.

**Results:**

The algorithm had an overall classification accuracy of 79.9% [sensitivity: 82.8, 95% CI (0.73–0.89), specificity: 77.0, 95% CI (0.67–0.85)]. The algorithm remained highly sensitive across subgroups stratified by age, sex, depression severity, comorbid psychiatric illness, cardiovascular disease, and smoking status.

**Conclusions:**

Sleep-derived heart rate patterns could act as an objective biomarker of depression, at least when it co-occurs with sleep disturbances, and may serve as a complimentary objective diagnostic tool. These findings highlight the extent to which some autonomic functions are impaired in individuals with depression, which warrants further investigation about potential underlying mechanisms.

**Electronic supplementary material:**

The online version of this article (10.1186/s12888-019-2152-1) contains supplementary material, which is available to authorized users.

## Introduction

The search for an objective biomarker of depression may lead to the development of complimentary clinical tools to improve diagnosis and reveal novel therapeutic targets. Changes in sleep physiology specifically linked to depressive states have been proposed as a candidate. As compared to healthy controls, people with depression have increased sleep latency, more fragmented sleep, a higher proportion of rapid eye movement (REM) sleep, decreased REM latency, and, in some cases, decreased amounts of slow-wave sleep (SWS) [1, 2]. However, these abnormalities in sleep architecture are not specific to depressive states [3–5]. In parallel to efforts investigating sleep-related characteristics of depression, research focused on identifying depression biomarkers in cardiovascular functions and related inflammatory processes offers promising findings, including increased levels of peripheral vascular endothelial growth factor [6, 7], interleukin-6 [8], and C-reactive protein [9]. Furthermore, depression is often accompanied by increased heart rate and reduced heart rate variability in both sleep and wake states [10, 11]. Considering the growing body of evidence suggesting that sleep disturbances may play an active role in the pathophysiology of both cardiovascular dysfunctions and depression, we propose that changes in heart rate during sleep may have potential as an objective biomarker of depression.

Approximately 80% of individuals with a current major depressive episode have co-occurring sleep difficulties [12], and sleep disruptions are known to be detrimental to cardiovascular function. Sleep loss can contribute to the emergence and/or worsening of cardiovascular ailments through changes in vascular functions [13], altered autonomic neuro-cardiac modulation [14], as well as dysregulation of the hypothalamic-pituitary axis [15]. For instance, experimental studies have demonstrated that sleep deprivation actively increases heart rate and plasma levels of vascular endothelial cell activation markers [13, 16]. Sleep deprivation also induces a shift in the autonomic sympathovagal balance towards increased sympathetic cardiac modulation [14], which may lead to alterations in cardiovascular regulatory processes. Thus, sleep difficulties linked to depression may influence cardiovascular functions.

The pathophysiological changes in both cardiovascular regulation and sleep patterns associated with depression are likely to interact and alter heart rate dynamics during sleep, thereby providing a relevant window to observe multi-systemic biomarkers of depression. The cardiovascular signature of depression may be more prominent during sleep since this state is shielded from several external influences such as fluctuating daytime stress, physical activity, and cognitive and emotional processing. Additionally, the reactivity of heart rate to endogenous dynamic changes across the night (e.g., progression of sleep stages, interactions between homeostatic and circadian processes, and changes in autonomic regulation) may yield more specific indices of depression. Previous findings indeed suggest that heart rate patterns across wake and sleep differ between people with depression, anxiety disorders and healthy controls [17]. In a subsequent study, Iverson, Stampfer, et al. (2002) reported an inter-rater agreement of 78% between two experts who visually inspected heart rate patterns across wake and sleep to determine the likelihood that an individual has a psychiatric illness or not [18]. While promising, this approach is subjective and time consuming. Furthermore, these previous studies were mostly descriptive and did not directly assess the validity of heart rate-based classifications against diagnoses determined by standard psychiatric assessment. Therefore, the current study aimed to validate the diagnostic accuracy of an automated heart rate profiling tool designed to capture changes across sleep-wake states in order to objectively classify individuals according to depression status. To assess generalizability, we also sought to compare classification performance across different subgroups based on potential confounding factors, including age, depression severity, psychiatric comorbidity, psychoactive and cardiovascular medication use, cardiovascular disease, and smoking status.

## Methods

An artificial intelligence based algorithm for automated heart rate profiling was developed by an industry-based team of scientists and engineers (Medibio Limited, VIC, Australia; Related patents: PCT/AU2016/050490 and PCT/AU2018/050578). This algorithm was modeled using machine-learning based on a sample of 1203 polysomnography recordings in people with clinician-based depression diagnoses and healthy controls (training sample), and then tested in a distinct sample of 174 cases (testing sample). Both the training and testing samples were collated by the authors from retrospective and secondary databases as described below. This project was approved by the Royal Ottawa Mental Health Centre (ROMHC) Group Research Ethics Board and the “Comité d’éthique de la recherche” of the Hôpital du Sacré-Cœur de Montréal. Data from all other sites was originally collected under approval from each site’s respective research ethics board. Preliminary analyses were previously done by the authors and an independent biostatistician in parallel on a slightly smaller portion of this sample for the purpose of CE marking (“Conformité Européenne”; Certificate Registration Number: 532495 MR6). In the present article, a larger data sample was used.

### Cases

#### Training sample

##### Depression group

The training sample contained 664 depression cases retrospectively collated from the ROMHC Sleep Disorders Clinic polysomnography recordings. All of these individuals were referred by physicians to the Sleep Disorders Clinic due to a sleep complaint such as insomnia, excessive daytime fatigue, or sleep-related breathing disturbances. To be included in this sample, all individuals had a diagnosis of a depressive syndrome documented in medical records (e.g., major depression, dysthymic disorder, depressive disorder not otherwise specified), and current depressive symptoms on the Beck Depression Inventory (BDI-II ≥14) [19] at the time of polysomnography. A psychologist reviewed and classified all diagnostic information for the purpose of this study. Table 1 provides the rates of psychiatric comorbidities, but none of the individuals included in this sample had a diagnosis of bipolar disorder or psychotic disorder.

**Table 1:** Psychiatric comorbidities in the depression training and testing samples

	Training Sample *n* (%)	Testing Sample *n* (%)
Any psychiatric comorbidity	240 (36.1)	29 (33.3)
Anxiety disorder	175 (26.4)	21 (24.1)
Post-traumatic stress disorder	3 (0.5)	0 (0.0)
Obsessive compulsive disorder	15 (2.3)	4 (4.6)
Personality disorder	31 (4.7)	4 (4.6)
Substance abuse disorder	54 (8.1)	8 (9.2)
Intermittent explosive disorder	0 (0.0)	1 (1.1)
Adjustment disorder	5 (0.8)	0 (0.0)
Eating disorder	13 (2.0)	0 (0.0)
Developmental disorder	33 (5.0)	5 (5.7)
Neurocognitive disorder	5 (0.8)	0 (0.0)

##### Control group

The training sample contained 529 healthy control cases. This sample was pooled from the National Sleep Research Resource (NSRR) [20–23] and the Montreal Archive of Sleep Studies (MASS) [24] databases. Control cases across both sites had no history of depression, anxiety disorder, ADHD, neurological disorders or sleep disorders. All cases from the MASS also scored asymptomatic on the BDI (BDI-II < 14 or BDI-Short Form < 5).

#### Testing sample

##### Depression group

The depression cases used for the final testing sample comprised a total of 87 individuals. These cases were issued from the ROMHC Sleep Disorders Clinic database, but were all distinct from the cases included in the training sample. Inclusion/exclusion criteria matched those of the training sample depression group. Additionally, in order to limit the heterogeneity of the sample, none of the patients included in this sample were taking antipsychotic medications or had a diagnosis of post-traumatic stress disorder. These additional exclusion criteria were applied on the remaining sample available for the testing phase.

##### Control group

The control group for the testing sample consisted of a total of 87 cases, all distinct from the control training sample. This sample was collated from four sites: the MASS (51 cases), Western University’s Brain & Mind Institute Sleep Research Laboratory (BMISRL; 20 cases), the “Centre d’étude des troubles du sommeil” (CÉTS: 3 cases), and the ROMHC Sleep Disorders Clinic (13 cases). Control cases sourced from the MASS, BMISRL and CÉTS where healthy controls undergoing standard polysomnography without any active intervention, and those from ROMHC Sleep Disorders Clinic were patients referred by physicians for the assessment of sleep abnormalities, including sleep-related breathing disturbances. Control cases were selected from larger datasets collected at each site in an effort to approximate the age and sex distribution of the depression cases. Inclusion/exclusion criteria matched those of the training sample control group. In addition, individuals from the MASS, BMISRL and CÉTS were excluded if they were using any medications known to interfere with sleep, participated in regular night work, or had been on a trans-meridian trip within 3 months prior to the polysomnography recording.

### Polysomnographic recordings

Polysomnography recordings were conducted with similar procedures at all sites. This included scalp electroencephalogram (EEG) channels F3 and/or Fz, C3 and/or Cz, and O1 and/or Oz, left and right electrooculogram (EOG), electrocardiogram (ECG), chin and leg electromyogram (EMG), blood oxygen saturation via an oximeter probe on the finger, and respiratory effort via a nasal thermistor, as well as thoracic and abdominal respiratory belts (MASS and ROMHC). Detailed acquisition parameters for each site are shown in Table 2. Sleep stage scoring was manually performed by qualified personnel in accordance with the clinical scoring guidelines established by the American Academy of Sleep Medicine [25]. Across all sites, none of the scorers were aware of the present study aims. The ECG signal was processed to remove artefacts and extract inter-beat interval (IBI) time series. In the testing set, all ECG recordings began at least 3 min prior to the first 30-s epoch scored as sleep (mean recording durations before and after sleep are reported in Table 3).

**Table 2:** EEG and ECG acquisition parameters

Site	Hardware (Software)	Sampling Rate (Filters) [Hz]	Timing of lights on/off	ECG Location
ROMHC	Sandman SD32 (Sandman 10.1; Natus Medical Incorporated, San Carlos, CA, USA)	256 (none)	Mean habitual bedtime and wake time (up to 6:15–6:30 AM)	Top of shoulders
NSRR	Edentrace I or II (Edentec, Eden Prairie, MN, USA)	128 (0.15)	Habitual sleep schedule	1) Below the midpoint of the right clavicle 2) Below the left breast crease, in line with the midpoint of the left clavicle (or below the midpoint of the left clavicle)
MASS	Grass 15A54 (Harmonie Stellate System; Natus Medical Incorporated, San Carlos, CA, USA)	256 or 512 (0.3–100)	Mean habitual bedtime and wake time	Left and right arms
BMISRL	Embla Titanium (RemLogic; Natus Medical Incorporated, San Carlos, CA, USA)	512 (0.1–220)	Mean habitual bedtime and wake time	Below each clavicle
CÉTS	Grass Model 12 (Harmonie Stellate System; Natus Medical Incorporated, San Carlos, CA, USA)	256 (0.01–100)	Mean habitual bedtime and wake time	Below each clavicle

*ROMHC* Royal Ottawa Mental Health Centre, *NSRR* National Sleep Research Resource, *MASS* Montreal Archive of Sleep Studies, *BMISRL* Western University’s Brain & Mind Institute Sleep Research Laboratory, *CÉTS* Centre d’étude des troubles du sommeil

**Table 3:** Sleep architecture and descriptive information for the testing sample

Variable	Control [x̅ ± SD]^b^	MDD [x̅ ± SD]^b^	*U*/*χ*^*2*^	*p*
*n*	87	87	–	–
Sex Distribution (%female)	55.2	58.6	.211	.646
Age (years)	43.3 ± 11.3	43.6 ± 10.5	3744.0	.903
AHI	1.7 ± 2.8	3.3 ± 5.0	2769.5	.002
BMI (kg/m^2^)	–	29.6 ± 6.8	–	–
BDI II Score	–	24.6 ± 9.0	–	–
Recording Time Before Sleep (min)	21.6 ± 15.0	40.2 ± 28.3	1882.5	.000
Recording Time After Sleep (min)	9.5 ± 28.9	4.6 ± 8.2	3398.5	.295
Total Recording Duration (min)	468.4 ± 35.6	474.2 ± 55.4	3550.5	.563
Total Sleep Time [TST (min)]	386.2 ± 43.8	355.6 ± 76.4	2891.0	.007
Sleep Efficiency (%)	87.7 ± 7.3	82.9 ± 14.3	3305.0	.149
Sleep Onset Latency (min)	13.6 ± 13.0	20.3 ± 18.7	2782.5	.003
REM Latency (min)^a^	98.6 ± 48.3	190.9 ± 106.6	1363.5	<.001
WASO (min)	55.1 ± 32.4	72.9 ± 62.2	3451.5	.316
N1 (min)	32.8 ± 18.8	66.4 ± 37.4	1617.5	<.001
N2 (min)	223.1 ± 47.9	207.3 ± 63.4	3151.0	.057
N3 (min)	54.7 ± 39.1	34.8 ± 33.7	2595.0	<.001
REM (min)	75.7 ± 25.5	47.1 ± 32.9	1788.0	<.001
N1 (% of TST)	8.7 ± 5.3	19.5 ± 11.5	1441.0	<.001
N2 (% of TST)	57.7 ± 10.5	58.0 ± 11.5	3760.0	.941
N3 (% of TST)	14.2 ± 10.1	9.7 ± 8.6	2758.0	.002
REM (% of TST)	19.4 ± 5.7	12.9 ± 9.0	1780.0	<.001

^a^Eight individuals from the depression group had no REM sleep ^b^Despite the use of a non-parametric test, the mean and standard deviation is reported for better clarity

*AHI* Apnea-Hypopnea Index, *BMI* Body Mass Index, *BDI* Beck Depression Inventory, *REM* Rapid eye movement, *WASO* Wake after sleep-onset, *N1* Non-REM 1, *N2* Non-REM 2, *N3* Non-REM 3

### Algorithm training and testing procedures

#### Data division

While collating the data sets, the independent research team used a random list generator to progressively select 10% of the data to put aside for the testing sample. The remainder of the data was used for algorithm training.

#### Features

The heart rate profiling algorithm is based on a computational model of the relationship between mental state and heart rate pattern characteristics. This integrated multiple features of ECG dynamics including heart rate and heart rate variability, as well as sleep stages scored from the EEG.

#### Training

The Medibio team was provided with a sample of de-identified ECG and EEG data including healthy controls and people with depression. Using this training sample, a logistic regression with lasso regularization model was employed to determine the optimal weight of each feature in order to attain the best classification of cases in the depression and control groups. To avoid over-fitting and over-optimistic results, a 10-fold cross-validation procedure was performed using the training sample.

#### Testing

All files from the testing sample were carefully reprocessed by the authors to ensure all formats and parameters were exactly the same across files from all sites (e.g. using the same labels and formats for derivations and sleep stage, excluding all signals except the ECG signals, down-sampling the ECG signal to the lowest acquisition rate). These files were identified by unique codes randomly allocated across the mixed sample of depression and control cases. The final algorithm was applied to this testing sample by the Medibio team under blinded conditions. The resulting classifications were sent back to the authors, who then conducted the independent validation analyses by comparing these to actual medical record diagnoses.

### Statistical analyses

All statistical analyses were carried out using the Statistical Package for the Social Sciences (SPSS, version 22.0, Armonk, NY: IBM Corp). For descriptive purposes, chi-square and Mann-Whitney U tests were performed in the final testing sample to detect any significant differences between the control and depression groups in the sex distribution, age, apnea/hypopnea index (AHI) and sleep architecture.

As part of the validation analyses, the diagnostic classification based on the heart rate profiling algorithm was compared to diagnostic information collated from medical records. This was done using a confusion matrix [26], as well as Cohen’s kappa statistic to determine ‘inter-rater agreement’ between the two sets of classifications.

To evaluate how potential confounders may affect the algorithm’s performance, the entire sample was stratified by age and sex to compare kappa statistics across subgroups. The depression group was further stratified according to Body Mass Index [Underweight (< 18.5 kg/m^2^), Normal (18.5 to 24.99 kg/m^2^), Overweight (25.0 to 29.99 kg/m^2^), and Obese (≥30 kg/m^2^) [27], psychotropic medication use, depression severity [Mild Depression (BDI- II: 14–19), Moderate Depression (BDI- II: 20–28), Severe Depression (BDI- II: 29–63)], the presence of psychiatric comorbidities, the presence of cardiovascular diseases or related risk factors, current smoking status and polysomnographic variables found to differ between the control and depression group (median split). The algorithm’s sensitivity was computed for each stratified subgroup.

Sleep architecture variables were compared for individuals who were incorrectly classified by the algorithm (i.e. false positives and false negatives) with age and sex-matched subsets of correctly classified individuals (i.e. true negatives and true positives). Furthermore, false positive rates were compared across all control sites in order to determine whether the use of different ECG systems had an impact on the algorithm’s performance.

## Results

### Descriptive group characteristics

The training sample depression group was comprised of 72.9% females (mean age = 45.0 ± 15.7 years; age range 14 to 85 years). Of the overall training sample depression group, 81.6% was taking psychotropic medication (see Table 4 for details). The sex distribution of the training sample control group was 55% female (mean age = 41.3 ± 18.5 years; age range 14 to 81 years). In the testing sample, the depression group was comprised of 59% females (mean age = 43.6 ± 10.5 years; age range = 20 to 71 years), and the control group was comprised of 55% females (mean age = 43.3 ± 11.3 years; age range = 20 to 65 years). Further descriptive information for both groups of the final testing sample is outlined in Table 3. There were no significant age or sex differences between the depression and control group. On average, total sleep time was 30.6 min shorter in the depression group than in the control group. Compared to the control group, the depression group also had significantly longer sleep onset latency and REM onset latency, as well as a lower amount of REM sleep, both in absolute time and as a percentage of total sleep time. Although the AHI remained below the clinical threshold for both groups, the depression group had a slightly but significantly higher AHI than the control group.

**Table 4:** Psychotropic medications in the depression training and testing samples

	Training sample *n* (%)	Testing sample *n* (%)
Any psychotropic medication	542 (81.6)	69 (79.3)
SSRI	239 (36.0)	28 (32.2)
SNRI	190 (28.6)	19 (21.8)
TCA	39 (5.9)	6 (6.9)
NaSSA	43 (6.5)	2 (2.3)
MAOI	4 (0.6)	1 (1.1)
NRI/NARI	5 (0.8)	1 (1.1)
SARI	119 (17.9)	25 (28.7)
NDRI	89 (13.4)	12 (13.8)
RIMA	6 (0.9)	1 (1.1)
Other antidepressant	0 (0.0)	0 (0.0)
Antipsychotic	126 (19.0)	0 (0.0)
Mood stabilizer	71 (10.7)	8 (9.2)
Lithium	17 (2.6)	3 (3.4)
Melatonin agent	15 (2.3)	1 (1.1)
Benzodiazepine	96 (14.5)	8 (9.2)
Hypnotic/Sedative/Anxiolytic	123 (18.5)	2 (2.3)
Stimulant	38 (5.7)	4 (4.6)
Dopamine agonist	1 (0.2)	3 (3.4)
NMDA receptor antagonist	0 (0.0)	1 (1.1)
Acetylcholinesterase inhibitor	1 (0.2)	0 (0.0)

*SSRI* Selective Serotonin Reuptake Inhibitor, *SNRI* Serotonin-Norepinephrine Reuptake Inhibitor, *TCA* Tricyclic Antidepressant, *NaSSA* Noradrenergic/Specific Serotonergic Antidepressant, *MAOI* Monoamine Oxidase Inhibitor, *NRI/NARI* Noradrenergic Reuptake Inhibitor, *SARI* Serotonin Antagonist and Reuptake Inhibitor, *NDRI* Norepinephrine-Dopamine Reuptake Inhibitor, *RIMA* Reversible Monoamine Oxidase Inhibitor

### Validation of the heart rate profiling algorithm

The classification matrix is shown in Table 5. Overall, the heart rate profiling algorithm had a classification accuracy of 79.9% [sensitivity: 82.8, 95% CI (0.73 to 0.89), specificity: 77.0, 95% CI (0.67 to 0.85)]. There was a moderate level of agreement with diagnoses derived from medical records [κ = 0.60, 95% CI (0.48 to 0.72), *p* < .001]. The degree of concordance between heart rate based classifications and diagnoses documented from medical records stratified by age and sex subgroups is shown in Table 6. Cohen’s kappa reached at least the moderate level in all subgroups, with similar concordance for males and females, and a higher concordance for the 36–50 year old subgroup.

**Table 5:** Confusion matrix of algorithm classification vs. actual diagnosis

	Actual Diagnosis			
	Depression	Control		
Algorithm			Total	
Depression	72^a^	20^b^	87	PPV: 78.3%
Control	15^c^	67^d^	87	FOR: 18.3%
Total	82	92	174	
	Sensitivity: 82.8%	FPR: 23.0%		
	FNR: 17.2%	Specificity: 77.0%		

^a^True positives: Individuals with depression who were correctly classified ^b^False positives: Healthy controls who were incorrectly classified as depressed^c^False negatives: Individuals with depression who were incorrectly classified as healthy controls ^d^True negatives: Healthy controls who were correctly classified

*PPV* Positive Predictive Value, *FOR* False Omission Rate, *FNR* False Negative Rate, False Positive Rate

**Table 6:** Classification agreement across age and sex groups in the testing sample

	Group	Cohen’s kappa (*κ*)^a^	*n*	95% CI	*p*
Sex	Female	0.60	99	0.44 to 0.75	<.001
	Male	0.60	75	0.42 to 0.78	<.001
Age Group	18–35	0.56	47	0.32 to 0.80	<.001
	36–50	0.72	71	0.54 to 0.89	<.001
	51–71	0.41	56	0.17 to 0.65	.002

^a^Concordance between heart rate-based classification and diagnoses documented from medical records

### Sensitivity stratifications

Table 7 lists the algorithm’s sensitivity across subgroups of the depression sample stratified by potential confounding factors. There was no significant difference in the algorithm’s sensitivity across subgroups based on psychiatric comorbidities, smoking status, the presence of cardiovascular disease and/or related risk factor, or the use of cardiovascular medication. Cardiovascular medication use and comorbid medical disorders, including cardiovascular diseases, are outlined for all groups in Additional file 1: Table S1 and Additional file 2: Table S2, respectively. Conversely, the algorithm demonstrated significant differences in sensitivity across subgroups stratified by psychotropic medication use (*χ*^*2*^ (1) = 4.1, *p* < .050) and body mass index (*χ*^*2*^(2) = 10.5, *p* = .005), with the highest sensitivities in the subgroup taking psychotropic medication, and the subgroup with a BMI in the obese range.

**Table 7:** Algorithm sensitivity across the testing sample depression group stratified by potential confounding factors

	Stratification	Sensitivity (%)^a^	*n*	*χ* ^*2*^(df)	*p*
Body Mass Index	Normal	61.9	21	10.5 (2)	.005
	Overweight	83.3	36		
	Obese	96.7	30		
Psychotropic Medication Use	Yes	87.0	69	4.1 (1)	<.050
	No	66.7	18		
Depression Severity	Mild	83.9	31	0.2 (2)	.927
	Moderate	80.6	31		
	Severe	84.0	25		
Psychiatric Comorbidity	Yes	75.9	29	1.5 (1)	.229
	No	86.2	58		
Cardiovascular Disease or Related Risk Factor	Yes	83.9	31	0.0 (1)	.838
	No	82.1	56		
Cardiovascular Medication Use	Yes	82.6	23	0.0 (1)	.982
	No	82.8	64		
Smoking Status	Non-Smoker	82.5	63	0.0 (1)	.930
	Current Smoker	83.3	24		
AHI	< 5	79.7	69	2.2 (1)	.141
	≥ 5	94.4	18		
Sleep Onset Latency (min)	≤ 15	82.2	45	0.0 (1)	.891
	> 15	83.3	42		
REM Onset Latency (min)	≤ 164	80.0	40	0.7 (1)	.390
	> 164	87.2	39		
%REM	≤ 13	90.9	44	4.1 (1)	<.050
	> 13	74.4	43		

^a^The algorithm’s ability to accurately detect cases of depression (i.e. true positive rate)

*AHI* Apnea-Hypopnea Index, *REM* Rapid eye movement

### False positives/false negatives

Fifteen of the 87 individuals with depression were incorrectly classified as controls (i.e. false negative: 60% female, mean age ± SD = 43.2 ± 14.3 years; age range = 20 to 68 years). Compared to age and sex-matched depression cases who were correctly classified, the false negative group had significantly lower amounts of N1 sleep in absolute time (t (28) = − 4.4, *p* < .001), with an average of 30.1 (± 13.7) minutes of N1 sleep, as compared to 60.4 (± 22.8) minutes of N1 sleep in the true positive group. There was no significant difference in AHI between false negatives (median = 0.6) and true positives (median = 2.7), *U* = 67.5, *p* = .061, nor between false positives (median = 0.7) and true negatives (median = 0.5), *U* = 186.0, *p* = .703.

Twenty of the 87 healthy controls were incorrectly classified as depressed (i.e. false positive: 55% female, mean age ± SD = 44.3 ± 12.1 years; age range = 20 to 63 years). Compared to age and sex-matched controls who were correctly classified, these false positives had significantly higher amounts of N1 (t (38) = 3.5, *p* = .001) and REM (t (38) = − 2.1, *p* < .05) sleep, in absolute time. The false positive group had an average of 45.6 (± 16.9) minutes of N1 sleep, and 65.4 (± 26.0) minutes of REM sleep, whereas the true negative group had an average of 27.0 (± 17.0) minutes of N1 sleep, and 81.5 (± 22.5) minutes of REM sleep. There were no significant differences in the rates of false positives across the BMISRL, CÉTS, MASS, and ROMHC sites (*x*^2^ (3) = 6.8, *p* = .080).

## Discussion

This study demonstrates, for the first time, that changes in heart rate across sleep-wake states may be valid physiological markers for the identification of depression in a sample of people with sleep complaints. The heart rate profiling algorithm classified individuals with an accuracy of 79.9%. Specifically, the algorithm was able to detect 82.8% of the depression cases, and rule out 77.0% of healthy controls (these results are in line with the preliminary analyses conducted for CE marking). In comparison, the detection rate of depression amongst primary care practitioners is thought to be approximately 47% [28]. Considering that the algorithm performed substantially well in comparison to standard diagnostic procedures, this tool may serve as an objective, adjunctive measure to assist in depression diagnosis and monitoring. Subsequent studies should evaluate whether currently accessible ambulatory ECG devices could be used to track sleep-derived profiles indicative of depression. From this perspective, the algorithm could potentially serve as a non-invasive and low-cost tool. The combined use of a classification instrument with clinician-based diagnosis may improve diagnostic accuracy and means of patient monitoring.

Depressive disorders are associated with sympathetic hyperactivity and reduced cardiac vagal control, which increases the risk of cardiovascular disease. Considering the associations between depression and inflammation [8, 9], as well as the role of the vagus nerve in mediating the cholinergic anti-inflammatory pathway [29, 30], it has been suggested that this autonomic imbalance may occur via the effects of pro-inflammatory cytokines on the central autonomic network [31]. The accuracy of this sleep ECG tool in detecting depression simply based on heart rate profiles during sleep highlights the extent to which autonomic neuro-cardiac regulation is dysfunctional in individuals with depression and sleep complaints. Further investigation is warranted to identify the specific physiological mechanisms of depression, alone or in combination with sleep disturbances, that underlie the abnormal profiles of heart rate dynamics during sleep. Given the increased risk of cardiac morbidity and mortality in these individuals, and the fact that increased heart rate and reduced heart rate variability are known to increase the risk of cardiac morbidity and mortality [32–34], this also emphasizes the need to assess whether chronic autonomic dysfunctions during sleep may, over time, contribute to poor health outcomes in people with depression. Prospective work is required to clarify how heart rate changes during sleep may relate to cardiovascular events and related risks factors in people with depression.

### Trait versus state marker

Preliminary studies of heart rate recordings during wake have shown that deviant heart rate patterns progressively normalize with effective antidepressant medication treatment of depression [35], and electroconvulsive therapy [36]. This reinforces the notion that the observed atypical heart rate patterns are manifestations of the physiological changes associated with depressive states resulting from autonomic nervous system dysfunction [37]. Whether heart rate profiles during sleep may be sensitive to changes in depressive states remains to be more thoroughly investigated. If further studies are able to establish that heart rate profiles during sleep normalize following successful treatment, the heart rate profiling algorithm used in this study may provide a helpful tool to objectively monitor treatment effectiveness over time.

### Sensitivity stratifications

The algorithm performed well across subgroups stratified based on sex, age, depression severity, comorbid psychiatric illness, comorbid cardiovascular disease and related risk factors, and smoking status. This suggests that the algorithm’s sensitivity to depression is generalizable across individuals with several potential confounding factors.

The algorithm had a significantly higher sensitivity in individuals with a BMI in the obese range and in those with elevated AHI, two factors often interconnected [38] and known to interact with cardiovascular functions. One the one hand, obesity is linked to a clear autonomic imbalance, with elevated sympathetic activity and lower vagal activity [39–41]. On the other hand, sleep-related breathing disorders are associated with a hyperactivation of the sympathetic nervous system due to intermittent hypoxia and sleep fragmentation [42]. Overall, cardiovascular alterations resulting from obesity and/or sleep-related breathing disturbances may have led to a higher likelihood of detection by the algorithm. Nevertheless, the algorithm’s sensitivity remained above 61 and 83% in people with a BMI within the normal and overweight ranges respectively, and remained above 79% in those with an AHI below 5. Even if the depression group had, on average, higher BMI and AHI than the control group, the ability of the algorithm to efficiently detect depression cases amongst individuals with low BMI and AHI suggests that the algorithm’s sensitivity to depression was not solely based on indirect effects of elevated BMI or AHI on the ECG.

The depression subgroup with psychotropic medication use also had a higher rate of correct detection based on the heart rate profiling algorithm. This could be simply due to differences in statistical power, as only 20.7% of the depression group was free of psychotropic medication use at the time of polysomnography. However, the use of antidepressants is associated with reductions in heart rate variability, and prospective studies are required to determine if these medications could possibly lead to better detection by the algorithm. Statistical power issues may also apply for the high detection rate in the 36–50 year age group, since this age bracket was over-represented. As such, the sensitivity of the algorithm should be further investigated in individuals who do not use psychotropic drugs, as well as across varying age groups. Various profiles of sleep disturbances have been found in individuals with depression, such as shortened REM onset latency, increased amounts of REM sleep as a percentage of total sleep time, increased REM density, and lengthened sleep onset latency [2, 4]. However, these abnormalities in REM sleep were not observed in our sample, possibly due to the use of antidepressant medication. However, in subgroups of patients with different sleep patterns, namely those with longer or shorter sleep, and longer or shorter REM onset latencies, the algorithm’s sensitivity remained consistent. Of note, the algorithm had a higher sensitivity in individuals with a lower proportion of REM sleep. Considering the higher classification sensitivity observed in those taking antidepressant medications, which are known to actively suppress REM sleep, this is likely to be related to the higher sensitivity found in the subgroup of medicated individuals. Interestingly, while the overall depression group had significantly higher amounts of N1 sleep on average than the controls, the subgroup of controls incorrectly classified as depression cases (i.e. false positives) also had high N1 amounts, and the depression cases incorrectly classified as controls (i.e. false negatives) had low N1 amounts. From this perspective, heart rate changes during shallow sleep may be an especially important feature used by the algorithm for the identification of depression.

Overall, the new biomarker developed herein persisted at a high level, independently of global differences in sleep architecture. Considering that abnormalities in standard EEG-based sleep measures are known to be poorly specific to depressive states [3–5], this multi-systemic biomarker tapping on dynamic changes in both brain and heart activity, may be more promising to distinguish between different mental illnesses.

## Limitations

We acknowledge several limitations in this study. Due to intellectual property rights, the specific content of the algorithm cannot be disclosed. The control group data was obtained from four different sites with slightly differing EEG and ECG acquisition parameters, while the depression group data was sourced from one site. However, the sourcing of data from multiple sites allowed testing the algorithm on a larger and more diverse sample, which highlights its robustness and generalizability. Also, there were no significant differences in the false positive rate across different sites. The depression group consisted of individuals with sleep complaints who were referred to a specialized sleep disorders clinic, but only a portion of the control group had sleep complaints and was referred to a sleep clinic. As such, the large majority of depression cases had significant sleep problems, including sleep-related breathing disorders, which may influence heart rate patterns. However, the average AHI amongst both testing groups were below the clinical cut-offs for sleep apnea. Although sleep disturbances often co-occur with depression, the sample used in the present study may not be representative of the general depression population. Further work is required to decipher the relative contribution of depression and sleep abnormalities, including breathing disturbances, to the abnormal heart profiles detected by the algorithm. Importantly, working with this sample allowed us to establish that the algorithm classification remained accurate across a wide range of sleep profiles. Moreover, due to the effects of psychotropic medication on cardiac activity, the use of psychotropic medication in the majority of our sample could possibly have facilitated better detection of depression by the algorithm. Therefore, the algorithm’s performance must be investigated in a larger sample of individuals free of psychotropic medication use. Due to the retrospective nature of this study, it was not possible to ascertain whether individuals from the depression group and part of the control group had traveled across time zones or worked night shifts close to the time of the sleep recordings.

## Conclusions

The current study validated a novel diagnostic classification tool based on an objective, multi-systemic biomarker of depression in a clinical sample of individuals with depression and sleep complaints. This tool, based on heart rate changes under the influence of autonomic regulation during sleep, was found to be highly generalizable across several potential confounding variables, as well as across differing ECG/EEG acquisition systems. In addition to providing an improved biological underpinning for the diagnosis of depression, this could possibly offer supplemental information to psychiatric clinical assessment, and objective measures for early screening. Moreover, the use of distinct physiological variables as biomarkers of depression may help emphasize the interactions between mental and physical health. This may contribute to reducing the stigma associated with depression, lifting some social barriers to accessing psychiatric treatment, and allowing for more holistic patient care.

## Additional files

Additional file 1: Cardiovascular medication use. (DOCX 15 kb)

Additional file 2: Comorbid medical disorders. (DOCX 17 kb)

## Data Availability

The ROMHC, BMISRL, and CÉTS datasets analyzed in the present study are not publicly available. The NSRR dataset analyzed in the present study is available from https://sleepdata.org/datasets The MASS data set analyzed in the present study is available from. https://massdb.herokuapp.com/en/

## Abbreviations

ACE: Angiotensin-converting enzyme
AHI: Apnea-hypopnea index
ARB: Angiotensin II receptor blocker
BDI: Beck depression inventory
BMI: Body mass index
BMISRL: Western University’s Brain & Mind Institute Sleep Research Laboratory
CE: Conformité Européenne
CÉTS: Centre d’étude des troubles du sommeil
ECG: Electrocardiogram
EEG: Electroencephalogram
EMG: Electromyogram
EOG: Electrooculogram
IBI: Inter-beat interval
MAOI: Monoamine oxidase inhibitor
MASS: Montreal Archive of Sleep Studies
N1: Non-REM 1
N2: Non-REM 2
N3: Non-REM 3
NaSSA: Noradrenergic/specific serotonergic antidepressant
NDRI: Norepinephrine-dopamine reuptake inhibitor
NRI/NARI: Noradrenergic reuptake inhibitor
NSRR: National Sleep Research Resource
OSA: Obstructive sleep apnea
REM: Rapid eye movement
RIMA: Reversible monoamine oxidase inhibitor
ROMHC: Royal Ottawa Mental Health Centre
SARI: Serotonin antagonist and reuptake inhibitor
SNRI: Serotonin-norepinephrine reuptake inhibitor
SSRI: Selective serotonin reuptake inhibitor
SWS: Slow-wave sleep
TCA: Tricyclic antidepressant
TST: Total sleep time
UARS: Upper airway resistance syndrome
WASO: Wake after sleep-onset
