# Supplementary material for: Using heart rate profiles during sleep as a biomarker of depression
Source: BMC Psychiatry. 2019 Jun 7;19:168. doi: 10.1186/s12888-019-2152-1 (PMC6554996; doi:10.1186/s12888-019-2152-1)
Supplement: Supplementary file 1 — Cardiovascular medication use. (DOCX 15 kb) [file 12888_2019_2152_MOESM1_ESM.docx]

**Supplemental Table 1. Cardiovascular medication use**

|  | **Training Sample** | | **Testing Sample** | |
| --- | --- | --- | --- | --- |
|  | **Depression  *n* (%)** | **Control  *n* (%)** | **Depression  *n* (%)** | **Control  *n* (%)** |
| Any Cardiovascular | 204 (30.7) | 69 (13.0) | 23 (26.4) | 5 (5.7) |
| Anticoagulants | 10 (1.5) | 4 (0.8) | 0 (0.0) | 0 (0.0) |
| Antiplatelet Agents | 65 (9.8) | 24 (4.5) | 9 (10.3) | 0 (0.0) |
| ACE Inhibitors | 36 (5.4) | 26 (4.9) | 9 (10.3) | 2 (2.3) |
| ARBs | 24 (3.6) | 0 (0.0) | 2 (2.3) | 1 (1.1) |
| Beta Blockers | 21 (3.2) | 13 (2.5) | 1 (1.1) | 1 (1.1) |
| Calcium Channel Blockers | 32 (4.8) | 14 (2.6) | 5 (5.7) | 0 (0.0) |
| Cholesterol Lowering | 106 (16.0) | 21 (4.0) | 8 (9.2) | 1 (1.1) |
| Diuretics | 32 (4.8) | 25 (4.7) | 5 (5.7) | 3 (3.4) |
| Digitalis glycosides | 0 (0.0) | 1 (0.2) | 0 (0.0) | 0 (0.0) |
| Vasodilators | 0 (0.0) | 0 (0.0) | 0 (0.0) | 0 (0.0) |
| Nitrates | 0 (0.0) | 1 (0.2) | 0 (0.0) | 0 (0.0) |
| Anti-diabetics | 54 (8.1) | 18 (3.4) | 6 (6.9) | 1 (1.1) |
| Other anti-hypertensives | 0 (0.0) | 6 (1.1) | 0 (0.0) | 0 (0.0) |
| Other anti-arrhythmic | 0 (0.0) | 1 (0.2) | 0 (0.0) | 0 (0.0) |

**ACE: Angiotensin converting enzyme, ARBs: Angiotensin II receptor blockers**
