# Supplementary material for: Using heart rate profiles during sleep as a biomarker of depression
Source: BMC Psychiatry. 2019 Jun 7;19:168. doi: 10.1186/s12888-019-2152-1 (PMC6554996; doi:10.1186/s12888-019-2152-1)
Supplement: Supplementary file 2 — Comorbid medical disorders. (DOCX 17 kb) [file 12888_2019_2152_MOESM2_ESM.docx]

**Supplemental Table 2. Comorbid medical disorders**

|  | **Training Sample** | | **Testing Sample** | |
| --- | --- | --- | --- | --- |
|  | **Depression *n* (%)** | **Control *n* (%)** | **Depression *n* (%)** | **Control *n* (%)** |
| **Any cardiovascular disorder** | **202 (38.0)^a^** | **81 (15.3)** | **31 (35.6)** | **3 (3.4)** |
| Hypotension | 2 (0.4) | 0 (0.0) | 0 (0.0) | 0 (0.0) |
| Orthostatic hypotension | 1 (0.2) | 0 (0.0) | 0 (0.0) | 0 (0.0) |
| Hypertension | 108 (20.3) | 50 (9.5) | 10 (11.5) | 3 (3.4) |
| Prehypertension | 1 (0.2) | 0 (0.0) | 1 (1.1) | 0 (0.0) |
| Obesity | 55 (10.4) | 0 (0.0) | 6 (6.9) | 0 (0.0) |
| Hypercholesterolemia | 46 (8.7) | 36 (6.8) | 3 (3.4) | 1 (1.1) |
| Type I diabetes | 5 (0.9) | 0 (0.0) | 2 (2.3) | 0 (0.0) |
| Type II diabetes | 50 (9.4) | 0 (0.0) | 6 (6.9) | 1 (1.1) |
| Gestational diabetes | 2 (0.4) | 0 (0.0) | 1 (1.1) | 0 (0.0) |
| Diabetes (unknown type) | 0 (0.0) | 23 (4.3) | 0 (0.0) | 0 (0.0) |
| Angina | 8 (1.5) | 4 (0.8) | 1 (1.1) | 0 (0.0) |
| Myocardial infarction | 15 (2.8) | 1 (0.2) | 1 (1.1) | 0 (0.0) |
| Stroke | 7 (1.3) | 0 (0.0) | 0 (0.0) | 0 (0.0) |
| Transient ischemic attack | 2 (0.4) | 0 (0.0) | 1 (1.1) | 0 (0.0) |
| Coronary artery disease | 25 (4.7) | 0 (0.0) | 1 (1.1) | 0 (0.0) |
| Congestive heart failure | 1 (0.2) | 1 (0.2) | 0 (0.0) | 0 (0.0) |
| Arrhythmia | 9 (1.7) | 13 (2.5) | 1 (1.1) | 0 (0.0) |
| Congenital heart defect | 0 (0.0) | 0 (0.0) | 1 (1.1) | 0 (0.0) |
| Cardiomyopathy | 3 (0.6) | 0 (0.0) | 0 (0.0) | 0 (0.0) |
| Left ventricular hypertrophy | 2 (0.4) | 0 (0.0) | 1 (1.1) | 0 (0.0) |
| Cardiac surgery | 13 (2.4) | 3 (0.6) | 3 (3.4) | 0 (0.0) |
| **Any sleep-related breathing disorder** | **558 (86.9)^b^** | **0 (0.0)** | **72 (86.7)^c^** | **10 (11.8)^d^** |
| Obstructive sleep apnea | 315 (49.0) | 0 (0.0) | 26 (31.3) | 6 (7.1) |
| Upper airway resistance syndrome | 184 (28.7) | 0 (0.0) | 34 (41.0) | 2 (2.4) |
| OSA & UARS | 59 (9.2) | 0 (0.0) | 12 (14.5) | 2 (2.4) |

**^a^ Cardiovascular Disorder data is missing for 133 participants in the Training Sample depression group**

**^b^ Sleep-Related Breathing Disorder data is missing for 22 participants in the Training Sample depression group
^c^ Sleep-Related Breathing Disorder data is missing for 4 participants in the Testing Sample depression group**

**^d^ Sleep-Related Breathing Disorder data is missing for 2 participants in the Testing Sample control group**
